# Compassion fatigue, work engagement, and psychological distress in health care workers treating patients with long COVID

**DOI:** 10.1002/pmrj.13383

**Published:** 2025-04-18

**Authors:** Hannah Uhlig‐Reche, Summer Rolin, Rasika Karnik, Maureen Lyons, Monica Verduzco‐Gutierrez

**Affiliations:** ^1^ Department of Rehabilitation Medicine UT Health San Antonio San Antonio Texas USA; ^2^ Department of Internal Medicine, Section General Internal Medicine University of Chicago Chicago Illinois USA; ^3^ Department of Medicine, Division of General Internal Medicine and Geriatrics Oregon Health & Science University Portland Oregon USA

## Abstract

**Background:**

Health care workers (HCWs) caring for patients with chronic disease are more likely to experience compassion fatigue. The impacts on HCWs caring for patients with a new complex chronic disease, long COVID, are unknown.

**Objective:**

To measure compassion fatigue, work engagement, and psychological distress in HCWs caring for patients with long COVID and investigate associations with personal history of long COVID and demographic and occupational characteristics.

**Design:**

Cross‐sectional survey study assessing demographic and occupational characteristics, Compassion Fatigue‐Short Scale (CF‐SS), Utrecht Work Engagement Scale‐3 (UWES‐3), and Screening Tool for Psychological Distress (STOP‐D) in HCWs caring for patients with long COVID.

**Setting:**

Online survey from March–June 2023.

**Participants:**

116 HCWs caring for patients with long COVID.

**Interventions:**

Not applicable.

**Main Outcome Measures:**

CF‐SS, UWES‐3, and STOP‐D scores and associations with personal history of long COVID, demographic and occupational factors.

**Results:**

HCWs with a personal history of long COVID had worse scores in all outcome measures compared to those without long COVID. Effects were moderate (UWES‐3: η^2^ = −0.09, *p* = .01; STOP‐D: η^2^ = 0.06, *p* = .02; CF‐SS: η^2^ = 0.07, *p* = .02). Outcome measures were comparable between physical medicine and rehabilitation physicians and other HCWs (*p* > .05). CF‐SS showed a small positive correlation (*r*_*s*_ = 0.19, *n* = 112, *p* = .04) with the percentage of the provider's patient population with long COVID. Mean outcome measures differed between career‐level groups with medium to large effects (UWES‐3: η^2^ = 0.13, STOP‐D: η^2^ = 0.06, CF‐SS: η^2^ = 0.06). Work engagement was worse in early career compared to late career (*p*  <.01). Psychological distress was worse in middle career compared to late career (*p* = .02). Compassion fatigue was worse in early career compared to late career (*p* = .02).

**Conclusions:**

Among HCWs caring for patients with long COVID, mean scores for all primary outcomes were worse in HCWs with a personal history of long COVID compared to those without. Early career HCWs caring for this population are less engaged and experience greater compassion fatigue whereas those in middle career experience greater psychological distress.

## INTRODUCTION

Compassion fatigue is a state of exhaustion and dysfunction resulting from prolonged exposure to secondary trauma.^1^ It can be a work‐related hazard in health care workers (HCWs) caring for patients experiencing stressful and/or traumatic events.^2^ Compassion fatigue differs from burnout. Burnout is an organizational hazard in difficult organizational environments whereas compassion fatigue is related to chronic secondary trauma or a single intensive event.^3^ Both may present with psychological symptoms and result in a negative impact on work.^4^ Increased work engagement and compassion can build individual resilience against both burnout and compassion fatigue and may have a positive impact on overall health, quality of life, and career longevity.^5^, ^6^

HCWs caring for patients with chronic disease have been found more likely to have exposure to secondary trauma and subsequent compassion fatigue, resulting in a decreased willingness to care for patients with chronic disease.^7^ A recent study of nurses in a large southwestern hospital system found that 46% of nurses reported moderate to high compassion fatigue, which was significantly associated with years of employment, job changes, and sick day use.^8^ In a 2023 international review, the prevalence of compassion fatigue among HCWs during the COVID‐19 pandemic was estimated to be >60%.^9^ Physicians reported higher levels of compassion fatigue compared to nurses in Spain and Italy during the pandemic.^10^, ^11^ In the wake of the COVID‐19 pandemic, patients are experiencing a new chronic disease state, long COVID, that may affect HCWs similar to the way caring for other chronic disease populations does. This may further exacerbate the compassion fatigue experienced during the pandemic.

Long COVID has recently been defined by the National Academies of Science, Engineering, and Medicine as “an infection‐associated chronic condition that occurs after SARS‐CoV‐2 infection and is present for at least 3 months as a continuous, relapsing and remitting, or progressive disease state that affects one or more organ systems.”^12^ Optimal medical management of long COVID is still under investigation and development and the patient needs are varied. A multidisciplinary approach is utilized with medical care often spearheaded by physical medicine and rehabilitation (PM&R) with a treatment team that may include pulmonology, cardiology, neurology, psychiatry, psychology, neuropsychology, physical therapy, occupational therapy, and speech and language pathology.^13^, ^14^, ^15^, ^16^, ^17^, ^18^ The scope of PM&R care aims to “enhance and restore functional ability and quality of life,” which is a broad objective relative to other physician counterparts.^19^ Many patients with long COVID have complex medical and social needs requiring an integrated care model that includes social work.^13^, ^14^ The majority of PM&R HCWs caring for this patient population are also completing disability paperwork.^14^ Burnout in physiatrists has been associated with lower perceived meaningfulness of physiatrist clinical work and worse integration of PM&R into clinical care.^20^ It is possible that HCWs caring for patients with long COVID may feel that the demands of this growing patient population take them away from their initial subspecialty and consequently affect their work engagement and psychological well‐being.

Investigating compassion fatigue, work engagement, and psychological distress in HCWs caring for patients with long COVID will allow for a better understanding of the ongoing impact of COVID‐19 on HCWs. During the first year of the COVID‐19 pandemic, global health care personnel were found to have an increased rate of burnout and compassion fatigue.^21^ As the symptoms of long COVID became more recognized, a 2021 cross‐sectional study of senior physician specialists in Ireland found that one quarter of respondents reported that they or a colleague had experienced long COVID, and most respondents screened positive for burnout.^22^ This study did not examine the rate of burnout in those with long COVID nor did it discern which of those HCWs provided care for patients with long COVID. A more recent systematic review of the literature assessing the effects of long COVID among HCWs found that those HCWs with long COVID struggled with their dual patient/doctor identity, experienced barriers to health care, and reported anxiety/depression in as many as 47% of study participants.^23^ To our knowledge, no published studies to date have examined compassion fatigue, work engagement, and psychological distress in HCWs caring for patients with long COVID.

The aim of this study is to (1) describe sociodemographic and occupational factors in HCWs caring for patients with long COVID; (2) measure compassion fatigue, work engagement, and psychological distress in these HCWs; (3) assess burnout in this group; and (4) evaluate the relationships of these metrics with sociodemographic and occupational factors, including a personal history of long COVID in the HCW. Understanding the scope of this issue may influence future organizational practices to implement or enhance factors that counteract compassion fatigue, reduce psychological distress, and improve work engagement thereby promoting career longevity in these HCWs.

## METHODS

A cross‐sectional anonymous survey study was conducted of HCWs directly caring for patients with long COVID. Non‐patient facing HCWs, such as those with purely administrative or teaching responsibilities, were not included. An online survey was administered via Qualtrics XM (Qualtrics, Provo, UT). The survey link was distributed to HCWs through the American Academy of Physical Medicine and Rehabilitation Post‐Acute Sequelae of SARS‐CoV‐2 Collaborative listserv and consent obtained. Participation was voluntary and did not involve compensation. This survey study was classified as exempt by the Institutional Review Board at the University of Texas Health Science at San Antonio.

Data collection included self‐reported demographics, clinical work descriptors, and personal history of long COVID. Occupational factors included work setting, physician versus nonphysician, practice specialty, career level, time spent in telehealth, percentage of patients treated for long COVID, clinic support, and if HCWs volunteered versus were instructed to see patients with long COVID. Validated self‐report questionnaires were administered to assess work engagement (Utrecht Work Engagement Scale‐3 [UWES‐3]), psychological distress (Screening Tool for Psychological Distress [STOP‐D]), and compassion fatigue (Compassion Fatigue‐Short Scale [CF‐SS]). UWES‐3 is an abbreviated version of UWES‐15. It is composed of three items ordinally scaled 0 (never) to 6 (always): feeling energy (vigor), enthusiasm (dedication), and immersion (absorption).^24^ Lower scores indicate less work engagement with scores 10 or higher suggestive of a high level of work engagement, with variations depending on the specific context and population.^25^ STOP‐D consists of five items that provide severity scores from 0 (not at all bothered) to 9 (severely bothered) for depression, anxiety, stress, anger, and low social support.^26^ Higher scores indicate more psychological distress with a score of at least 25 suggestive of psychological distress.^26^ It has been found to accurately identify depressed patients and have high specificity and sensitivity for screening for anxiety.^26^, ^27^ CF‐SS is a 13‐item questionnaire assessing two dimensions of burnout (eight items) and trauma (five items) on a 10‐point Likert scale from 1 (never) to 10 (always).^28^ Higher scores indicate more severe compassion fatigue. The literature suggests that scores >56 may indicate significant compassion fatigue.^29^ Respondents scoring at least one SD above the mean score on the CF‐SS burnout dimension were categorized into a “burnout” group for further analysis.

Descriptive statistics were calculated according to normality determined by Kolmogorov–Smirnov test. Means, SDs, ranges, and medians were calculated for quantitative variables. Counts and percentages were calculated for categorical variables. Given the differences in scales, which are not linear, a nonnormal distribution was predicted, thus nonparametric tests (Mann–Whitney *U* and Kruskal–Wallis H) were used to compare means by participants' sociodemographic and occupational characteristics. Pearson's chi‐square test was performed to test categorical variables. Spearman's rank correlation was used for the analysis of correlations between main variables. Stepwise multiple linear regression analysis was performed for each of the CF‐SS, STOP‐D, and UWES‐3 components. A one‐way analysis of variance between groups was conducted to explore the impact of career level on UWES‐3, STOP‐D, and CF‐SS scores. Effect size was calculated using eta squared. Sidak test was used for post‐hoc comparisons of mean scores.

## RESULTS

There were 116 respondents included in this study with the majority identifying as female (63.8%) and younger than 45 years of age (60.5%). Career level was identified as early (0–9 years) in 43.4% of respondents. Most were partnered (89.5%) and parents (70.2%). A majority (57.4%) were physicians, working in academic medicine (64.7%), working primarily in an outpatient setting (62.1%), working full time (93.1%), and salaried (89.7%). Physician specialists included PM&R, cardiology, family medicine, infectious disease, internal medicine, neurology, orthopedics, psychiatry, and pulmonology. Nonphysician HCWs included neuropsychologists, psychologists, physician assistants, nurse practitioners, nurses, medical assistants, physical/occupational/speech therapists, social workers, community health workers, and other master's level providers. Table 1 displays sociodemographic and occupational characteristics.

**TABLE 1:** Demographics and work‐related characteristics.

Descriptors of sample	*n*	%
Age range (years)		
18–24	1	0.9
25–34	25	21.9
35–44	43	37.7
45–54	17	14.9
55–64	23	20.2
65 and older	5	4.4
Gender		
Female	74	63.8
Male	41	35.3
Prefer not to say/other	1	0.9
Married		
Yes	102	89.5
No	9	7.9
Separated	1	0.9
Prefer not to say	2	1.7
Children		
Yes	80	70.2
No	34	29.8
Career level		
Early (0–9 years)	49	43.4
Middle (10–15 years)	30	26.5
Late (15 years +)	34	30.1
Primary work setting		
Academic medicine	75	64.7
Private hospital	8	6.9
Public hospital	12	10.3
Community clinic	12	10.3
Private practice	3	2.6
Veterans Affairs system	6	5.2
Specialty		
PM&R physician	37	32.2
Other physician^a^	29	25.2
Other provider^b^	49	42.6
% of time spent with telehealth		
≤10	60	52.2
20	27	23.5
≥30	28	24.3
% of patients treated for long COVID		
10	32	27.8
20	24	20.9
30	16	13.9
40	11	9.6
50	14	12.2
60	6	5.2
70	1	0.9
80	3	2.6
90	2	1.7
100	6	5.2
Volunteered to treat long COVID		
Yes	61	53.5
No	53	46.5
Providers with long COVID		
With long COVID	19	22.1
Without long COVID	67	77.9

*Note*: age range, married, children, volunteers, *N* = 114; career level, *N* = 113; gender and work setting, *N* = 116; specialty, telehealth, and % of patients with long COVID, *N* = 115; Providers with long COVID, *N* = 86. Abbreviation: PM&R, physical medicine and rehabilitation.

^a^ This category included providers in cardiology, family medicine, infectious disease, internal medicine, neurology, orthopedics, psychiatry, and pulmonology.

^b^ This category included providers in neuropsychology, psychology, physician assistants, nurse practitioners, nurses, medical assistants, physical/occupational/speech therapists, social workers, community health workers, and other master's level providers.

The mean (SD) scores were 13.2 (3.4) on the UWES‐3, 15.4 (9.7) on the STOP‐D, and 40.3 (20.1) on CF‐SS. These mean scores do not meet the cutoff thresholds defined for work disengagement, psychological distress, or compassion fatigue; however, these thresholds are within one SD of the mean for all measures. There were no differences in outcome measures based on gender or marital or parental status.

Ten physicians were categorized into the burnout group and nine nonphysicians were categorized into the burnout group, which was not a statistically significant difference (physicians: 15.2%; nonphysicians: 18.0%; *X* ^2^ = 0.17, *p* = .68). Of physicians in the burnout group, five were in PM&R and five were in other medical specialties, a difference that was not statistically significant (PM&R: 13.5%; other medical specialties: 17.2%; *X* ^2^ = 0.18, *p* = .68).

Approximately 32% of total respondents were PM&R physicians and 56% of physician respondents were in PM&R. There was no difference in psychological distress scores for PM&R physicians compared to other respondents (PM&R: M = 13.3, SD = 9.0; Other respondents: M = 15.8, SD = 10.3; t (114) = −1.4, *p* = .16, two tailed). There was similarly no significant difference UWES‐3 for PM&R physicians (M = 12.7, SD = 3.9) compared to other providers (M = 13.6, SD = 3.2; *t* (113) = −1.4, *p* = .17, two tailed) or in compassion fatigue scores (PM&R: M = 37.9, SD = 20.5; other providers: M = 40.4, SD = 21.3; t (111) = −0.63, *p* = .53, two tailed).

**FIGURE 1:**
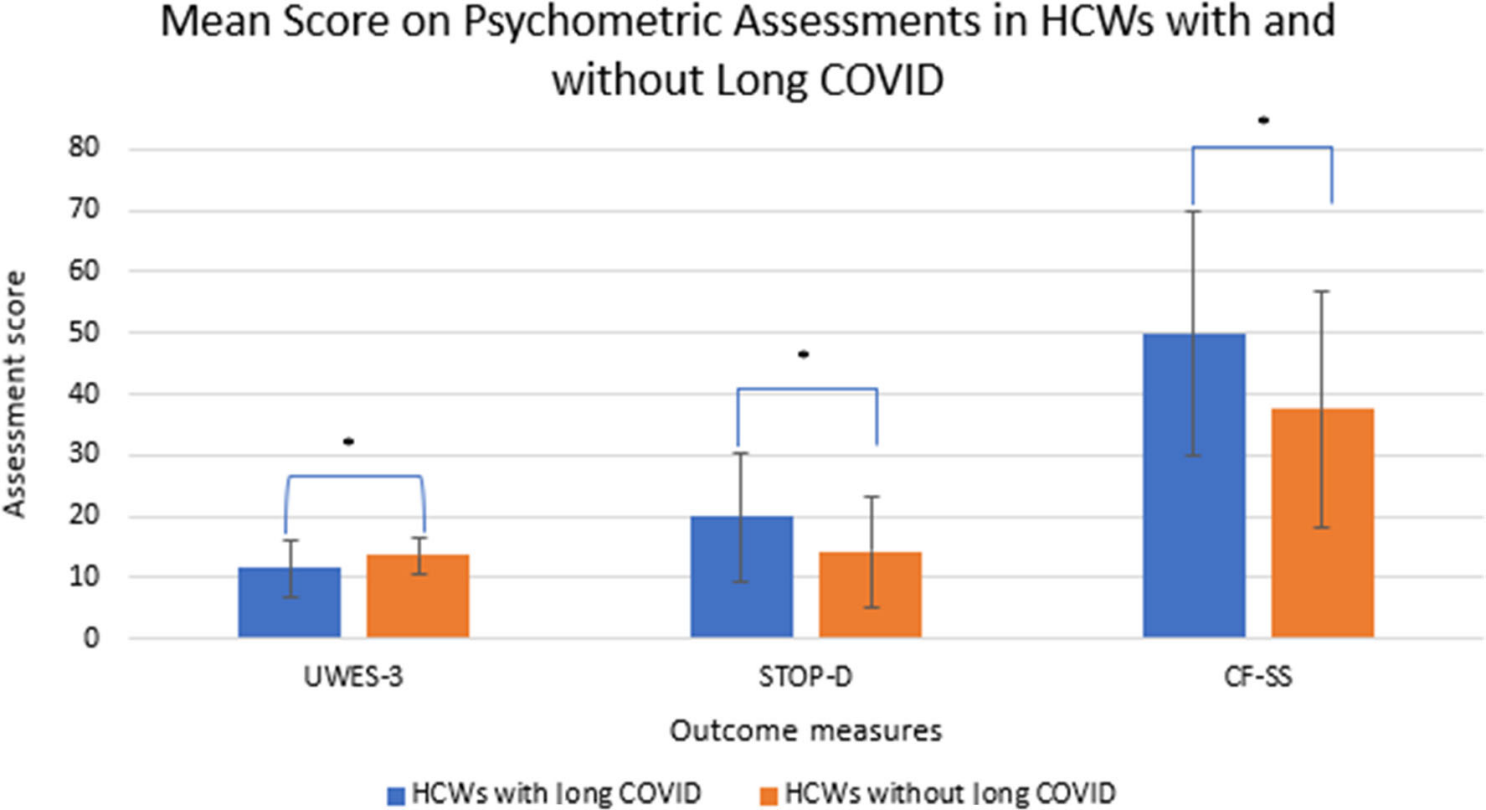
Mean score on psychometric assessments in HCWs caring for patients with long COVID who reported having long COVID compared to those who did not have long COVID. HCWs with long COVID reported statistically lower work engagement, greater psychological distress, and greater compassion fatigue than HCWs without long COVID. Error bars signify SD. * Denotes statistically significant differences at the *p* < .05 level. CF‐SS, Compassion Fatigue‐Short Scale; HCW, health care worker; STOP‐D, Screening Tool for Psychological Distress; UWES‐3, Utrecht Work Engagement Scale‐3.

There was a significant difference in all scores for providers with long COVID and without long COVID (Figure 1). UWES‐3 scores were significantly lower in providers with long COVID (M = 11.5, SD 4.5) than without (M = 13.7, SD = 2.9; t(84) = −2.60, *p* = .01, two tailed) with a moderate effect size (mean difference = −2.2, 95% confidence interval [CI] = −4.0 to −0.5, η^2^ = −0.09). STOP‐D scores were significantly higher in providers with long COVID (M = 19.9, SD = 10.5) than without (M = 14.2, SD = 9.2; *t* (85) = 2.3, *p* = .02, two tailed) with a moderate effect size (mean difference = 5.7, 95% CI = 0.8–10.6, η^2^ = 0.06). CF‐SS scores were significantly higher in patients with long COVID (M = 49.8, SD = 20.0) than without (M = 37.6, SD = 19.5; *t* (83) = 2.4, *p* = .02, two tailed) with a moderate effect size (mean difference = 12.2, 95% CI = 2.1–22.4, η^2^ = 0.07).

Outcome measures differed between career level groups with medium to large effects (UWES‐3: η^2^ = 0.13, STOP‐D: η^2^ = 0.06, CF‐SS: η^2^ = 0.06). There was a significant difference in UWES‐3 scores between career‐level groups (*p*  <.01). Post‐hoc comparisons indicated the mean score for the early career group (M = 12.0, SD = 3.8) was significantly lower than for the late career group (M = 14.9, SD = 2.2, *p* < .01). There was a significant difference in STOP‐D scores between career‐level groups (*p* = .03). Post‐hoc comparisons indicated the mean score for the middle career group (M = 40.8, SD = 22.3) was significantly higher than the late career group (M = 31.0, SD = 16.3, *p* = .02). There was a significant difference in CF‐SS scores between career‐level groups (*p* = .02). Post‐hoc comparisons indicated the mean score for the early career group (M = 43.1, SD = 20.8) was significantly higher than the late career hroup (M = 31.0, SD = 16.3, *p* = .02). The CF‐SS score showed a small positive correlation (*r*_*s*_ = 0.19, *n* = 112, *p* = .04) with the percentage of the provider's patient population with long COVID. There was not a significant difference in scores for providers who elected versus those who were mandated to care for patients with long COVID. There was also not a significant relationship between the outcome measure scores and type of clinic support. There was not a significant difference in scores relating to percentage of time delivering telehealth care. See Table 2 for associations of scores with these occupational factors.

**TABLE 2:** Associations with UWES‐3, STOP‐D, and CF‐SS scores and occupational factors.

Outcome	Clinic factor	*r*_*s*_	*t*	*n*	*p* value
UWES‐3					
	Percentage of long COVID patients	−0.01		112	.89
	Volunteer vs. Not		−0.53	114	.60
	Clinic support	0.04		115	.69
	Time spent in telehealth	−0.01		115	.27
STOP‐D					
	Percentage of long COVID patients	0.16		112	.07
	Volunteer vs. not		−1.48	115	.14
	Clinic support	0.00		116	.98
	Time spent in telehealth	0.03		112	.58
CF‐SS					
	Percentage of long COVID patients	0.19		112	.**04**
	Volunteer vs. not		−1.02	113	.31
	Clinic support	0.03		113	.68
	Time spent in telehealth	0.04		110	.85

*Note*: Clinic factors included in the table: percentage of all patients who are long COVID patients; providers volunteered to care for long COVID patients versus providers were assigned these patients; clinic support type available (onsite, offsite, clinic practice manager, administrative support, nurse coordinator, social work, behavioral health/psychology, specific clinic scheduling available); time spent in telehealth is indicative of the percentage of health care time spent in telehealth encounters (<10%, 10–29%, ≥30%).

Abbreviations: CF‐SS, Compassion Fatigue‐Short Scale; STOP‐D, Screening Tool for Psychological Distress; UWES‐3, Utrecht Work Engagement Scale‐3.

## DISCUSSION

This cross‐sectional survey study revealed that HCWs with a personal history of long COVID who are caring for patients with long COVID experience greater psychological distress, compassion fatigue, and work disengagement compared to those HCWs without a personal history of long COVID. Compassion fatigue is increased in HCWs caring for a higher percentage of patients with long COVID. Differences in these mental health outcome measures are also seen between HCW career‐level groups. Early career HCWs caring for this patient population are less engaged and experience greater compassion fatigue and those in middle career experience greater psychological distress.

In HCWs with long COVID, outcome scores indicate less work engagement, greater psychological distress, and greater compassion fatigue than HCWs without long COVID. Those HCWs with long COVID may experience mental health consequences associated with their underlying medical condition that is unrelated to their patient population at work.^30^ HCWs with long COVID may also have a lower baseline energy reserve to protect them from burnout, work disengagement, and compassion fatigue after exposure to the various demands associated with caring for patients with long COVID. They may experience their own challenges relating to their health condition or medical care, lowering their threshold to experience compassion fatigue while working to provide care to patients with long COVID.^31^ Despite the growing recognition of long COVID, there remains a shortage of both HCWs and specialized clinics equipped and available to treat patients and manage this complex condition.^14^, ^31^, ^32^, ^33^ A potential contributing factor to HCW distress is the current lack of resources for patients with long COVID nationally and the subsequent inability to provide the necessary care due to resource inaccessibility and lack of established protocols.^14^, ^23^

Results of this study show that treating a greater percentage of patients with long COVID is associated with increased compassion fatigue. It is plausible that the more patients with long COVID a HCW treats, the more likely they are exposed to the secondary trauma involved in the development of compassion fatigue. The psychological impact on HCWs caring for persons with long COVID, compounded by insufficient resources and limited access to specialized clinics, may contribute to a decrease in providers available or willing to treat persons with this chronic condition.^7^ This shortage is exacerbated by the overwhelming number of persons with long COVID in need of care, juxtaposed against the inadequate number of clinics and resources allocated to long COVID management nationwide. Because the optimal medical management of long COVID is still under investigation, the complexity of caring for this population introduces challenges that may be fatiguing.

In this study sample, early career HCWs caring for patients with long COVID are less engaged in their work and experience greater compassion fatigue and those in middle career experience more psychological distress. Those in early career may be more likely to have treated patients with acute COVID‐19 infection during the early pandemic due to recent residency training that may have required inpatient coverage of these patients. Thus, they may be at higher risk of burnout and compassion fatigue associated with the trauma of treating these acutely ill patients.^22^ Because long COVID is a relatively new chronic condition, early career HCWs may be less engaged in caring for this patient population because it was not an area of health care they initially envisioned in their career. HCWs in later career are likely to have a more established practice aligned with their original area of interest whereas early career HCWs may not experience this same sense of connection or consistency with their patient panel. Additionally, those in early career may not have a well‐established professional network from which to gather support and resources that foster work engagement and career development. Middle career HCWs may find themselves caring for patients with a novel chronic disease that they have not previously treated, which may be psychologically distressing due to the unfamiliarity with the pathologic process, whereas some early career HCWs may be more familiar with long COVID due to training during the pandemic. It is also possible that middle career HCWs progressed from compassion fatigue to psychological distress as compassion fatigue has been correlated with depression and anxiety.^34^ Late career HCWs may have developed successful coping strategies learned from experiencing challenges and psychological stressors in their earlier career.

Results of this study show that PM&R physicians caring for patients with long COVID experience comparable psychological distress, compassion fatigue, and work engagement scores to other HCWs caring for these patients. PM&R has emerged as a leader in the management of patients with long COVID and PM&R practitioners often serve as the primary providers managing this chronic condition, coordinating multidisciplinary care.^13^, ^14^, ^15^, ^16^, ^17^, ^18^ Formal medical training in PM&R did not include long COVID, as it is a newly recognized disease state, yet PM&R physicians are adept at addressing various symptoms as well as functional impairments and sequelae that affect patients' daily lives. The comparable outcome measures in PM&R physicians caring for this patient population is reassuring; however, nonresponse bias is a limitation that is recognized.

Future studies should investigate strategies to improve occupational well‐being in HCWs caring for patients with long COVID. A recent qualitative study in PM&R physicians highlighted the importance of personal lifestyle choices, work–life harmony, and strategies to improve professional satisfaction that may be applicable to this population of HCWs.^35^ There are likely organizational as well as personal strategies to protect against or recover from compassion fatigue, psychological distress, and work disengagement and thus combat burnout.^35^, ^36^, ^37^ Various organizational contexts have been identified that relate to improvement of work engagement, including fostering an organizational culture that focuses on person‐centered care rather than productivity metrics, management practices to overcome bureaucracy, and providing opportunities for employee professional development and self‐care.^35^, ^36^, ^37^

## LIMITATIONS

This study is limited by survey bias or nonresponse bias. Nonrespondents may not have participated due to the presence of work disengagement, psychological distress, or lack of time or energy. Another limitation is that the validated questionnaires used did not directly measure burnout, although burnout‐related questions were included particularly in the CF‐SS. STOP‐D is validated in measuring psychological distress in patients rather than HCWs, which may also be considered a limitation. The STOP‐D results may be confounded by factors external to occupation. Although a strength of this study is that it included HCWs in multiple roles, not just physicians, its generalizability is limited as some of the auxiliary roles are not well represented in respondents. Another limitation is that we were unable to compare the outcome measures in HCWs caring for patients with long COVID to HCWs not caring for this patient population.

## CONCLUSION

HCWs are vulnerable to psychological distress, compassion fatigue, and reduced work engagement post pandemic. Higher compassion fatigue is seen in those treating a greater percentage of patients with long COVID, likely due to an increased exposure to secondary trauma. HCWs who have long COVID and are also caring for persons with long COVID experience worse psychological distress, more compassion fatigue, and less work engagement compared to HCWs without long COVID. Early career HCWs caring for patients with long COVID are less engaged and experience greater compassion fatigue, and those in middle career experience greater psychological distress. It is crucial to prioritize the well‐being of the HCWs caring for this patient population by providing sufficient organizational support to combat compassion fatigue and psychological distress and promote work engagement. This includes access to mental health resources, fostering a culture focused on person‐centered care, and opportunities for employee professional development and self‐care. It is evident that supporting these HCWs is imperative, as their distress is likely linked to the current national deficiency in resources for patients with long COVID. Identifying and understanding the psychological distress that these HCWs may be experiencing is important to facilitate an improved experience and thus aid in the retention of these health services in a shortage area. By supporting HCWs caring for patients with long COVID, we can ensure sustainable health care delivery and promote a healthier workforce capable of meeting the ongoing challenges posed by this complex condition.

## DISCLOSURE

None.

## References

[1] Figley C . Compassion Fatigue. Coping with Secondary Traumatic Stress Disorder in Those Who Treat the Traumatized. Taylor & Francis Group; 1995.

[2] Munroe JF . Ethical Issues Associated with Secondary Trauma in Therapists. Secondary Traumatic Stress: Self‐care Issues for Clinicians, Researchers and Educators. Sidran Press; 1999:211‐229.

[3] Slatten LA , Carson KD , Carson PP . Compassion fatigue and burnout: what managers should know. Health Care Manag. 2011;30(4):325‐333. 10.1097/HCM.0b013e31823511f7 22042140

[4] Cao X , Chen L . The impact of resilience on turnover intention in dialysis nurses: the mediating effects of work engagement and compassion fatigue. Jpn J Nurs Sci. 2021;18(3):e12414. 10.1111/jjns.12414 33682287

[5] Chan AO , Chan YH , Chuang KP , Ng JSC , Neo PSH . Addressing physician quality of life: understanding the relationship between burnout, work engagement, compassion fatigue and satisfaction. J Hosp Admin. 2015;4(6):46‐55. doi:10.5430/jha.v4n6p46

[6] Rao S , Ferris TG , Hidrue MK , et al. Physician burnout, engagement and career satisfaction in a large academic medical practice. Clin Med Res. 2020;18(1):3‐10. doi:10.3121/cmr.2019.1516 31959669 PMC7153796

[7] Karabuga Yakar H , Oguz S , Bulut B , et al. Compassion fatigue in nurses caring for chronic diseases. Int J Occup Saf Ergon. 2023;29(1):109‐114. 34979885 10.1080/10803548.2021.2025314

[8] Wijdenes KL , Badger TA , Sheppard KG . Assessing compassion fatigue risk among nurses in a large urban trauma center. J Nurs Adm. 2019;49(1):19‐23. doi:10.1097/NNA.0000000000000702 30499866

[9] Garnett A , Hui L , Oleynikov C , Boamah S . Compassion fatigue in healthcare providers: a scoping review. BMC Health Serv Res. 2023;23(1):1336. doi:10.1186/s12913-023-10356-3 38041097 PMC10693134

[10] Carmassi C , Dell'Oste V , Bertelloni CA , et al. Gender and occupational role differences in work‐related post‐traumatic stress symptoms, burnout and global functioning in emergency healthcare workers. Intensive Crit Care Nurs. 2022;69:1‐7. doi:10.1016/j.iccn.2021.103154 34895972

[11] Ruiz‐Fernández MD , Ramos‐Pichardo JD , Ibáñez‐Masero O , Cabrera‐Troya J , Carmona‐Rega MI , Ortega‐Galán ÁM . Compassion fatigue, burnout, compassion satisfaction and perceived stress in healthcare professionals during the COVID‐19 health crisis in Spain. J Clin Nurs. 2020;29(21–22):4321‐4330. doi:10.1111/jocn.15469 32860287

[12] National Academies of Sciences, Engineering, and Medicine . A Long COVID Definition: A Chronic. Systemic Disease State with Profound Consequences. Washington, DC: The National Academies Press. 2024;10:17226/27768. doi:10.17226/27768 39110819

[13] Barshikar S , Laguerre M , Gordon P , Lopez M . Integrated care models for long coronavirus disease. Phys Med Rehabil Clin N Am. 2023;34(3):689‐700. doi:10.1016/j.pmr.2023.03.007 37419540 PMC10165471

[14] Dundumalla S , Barshikar S , Niehaus WN , Ambrose AF , Kim SY , Abramoff BA . A survey of dedicated PASC clinics: characteristics, barriers and spirit of collaboration. PM R. 2022;14(3):348‐356. doi:10.1002/pmrj.12766 35038230

[15] Whiteson JH , Azola A , Barry JT , et al. Multi‐disciplinary collaborative consensus guidance statement on the assessment and treatment of cardiovascular complications in patients with post‐acute sequelae of SARS‐CoV‐2 infection (PASC). PM&R. 2022;14(7):855‐878. 35657351 10.1002/pmrj.12859 PMC9347705

[16] Melamed E , Rydberg L , Ambrose AF , et al. Multidisciplinary collaborative consensus guidance statement on the assessment and treatment of neurologic sequelae in patients with post‐acute sequelae of SARS‐CoV‐2 infection (PASC). PM&R. 2023;15(5):640‐662. 36989078 10.1002/pmrj.12976

[17] Herrera JE , Niehaus WN , Whiteson J , et al. Multidisciplinary collaborative consensus guidance statement on the assessment and treatment of fatigue in postacute sequelae of SARS‐CoV‐2 infection (PASC) patients. PM&R. 2021;13(9):1027‐1043. 34346558 10.1002/pmrj.12684 PMC8441628

[18] Munipalli B , Seim L , Dawson NL , et al. Post‐acute sequelae of COVID‐19 (LONG COVID): a meta‐narrative review of pathophysiology, prevalence, and management. SN Compr Clin Med. 2022;4(1):90. 35402784 10.1007/s42399-022-01167-4 PMC8977184

[19] About Physical Medicine & Rehabilitation . www.aapmr.org. Accessed September 23, 2024. https://www.aapmr.org/about-physiatry/about-physical-medicine-rehabilitation.

[20] Makowski MS , Trockel M , Paganoni S , et al. Occupational characteristics associated with professional fulfillment and burnout among US physiatrists. Am J Phys Med Rehabil. 2023;102(5):379‐388. doi:10.1097/PHM.0000000000002216 37076955

[21] Lluch C , Galiana L , Doménech P , Sansó N . The impact of the COVID‐19 pandemic on burnout, compassion fatigue, and compassion satisfaction in healthcare personnel: a systematic review of the literature published during the first year of the pandemic. Healthcare Basel. 2022;10(2):364. doi:10.3390/healthcare10020364 35206978 PMC8872521

[22] Doherty AM , Colleran GC , Durcan L , Irvine AD , Barrett E . A pilot study of burnout and long covid in senior specialist doctors. Ir J Med Sci. 2022;191(1):133‐137. doi:10.1007/s11845-021-02594-3 33713306 PMC7955691

[23] Cruickshank M , Brazzelli M , Manson P , Torrance N , Grant A . What is the impact of long‐term COVID‐19 on workers in healthcare settings? A rapid systematic review of current evidence. PLoS One. 2024;19(3):e0299743. doi:10.1371/journal.pone.0299743 38442116 PMC10914278

[24] Merino‐Soto C , Lozano‐Huamán M , Lima‐Mendoza S , Calderón de la Cruz G , Juárez‐García A , Toledano‐Toledano F . Ultrashort version of the Utrecht Work Engagement Scale (UWES‐3): a psychometric assessment. Int J Environ Res Public Health. 2022;19(2):890. 35055713 10.3390/ijerph19020890 PMC8775405

[25] Schaufeli W , Schaufeli WB , Shimazu A , Hakanen J , Salanova M , De Witte H . An ultra‐short measure for work engagement. Eur J Psychol Assess. 2019;35(4):577‐591. doi:10.1027/1015-5759/a000430

[26] Young QR , Nguyen M , Roth S , Broadberry A , Mackay MH . Single‐item measures for depression and anxiety: validation of the screening tool for psychological distress in an inpatient cardiology setting. Eur J Cardiovasc Nurs. 2015;14(6):544‐551. 25139467 10.1177/1474515114548649

[27] Young QR , Ignaszewski A , Fofonoff D , Kaan A . Brief screen to identify 5 of the most common forms of psychosocial distress in cardiac patients: validation of the screening tool for psychological distress. J Cardiovasc Nurs. 2007;22(6):525‐534. doi:10.1097/01.JCN.0000297383.29250.14 18090195

[28] Adams RE , Boscarino JA , Figley CR . Compassion fatigue and psychological distress among social workers: a validation study. Am J Orthopsychiatry. 2006;76(1):103‐108. 16569133 10.1037/0002-9432.76.1.103 PMC2699394

[29] McGrath K , Matthews LR , Heard R . Predictors of compassion satisfaction and compassion fatigue in health care workers providing health and rehabilitation services in rural and remote locations: a scoping review. Aust J Rural Health. 2022;30(2):264‐280. doi:10.1111/ajr.12857 35267227 PMC9310831

[30] Walia N , Lat JO , Tariq R , et al. Post‐acute sequelae of COVID‐19 and the mental health implications. Discoveries (Craiova, Romania). 2021;9(4):e140. 35359346 10.15190/d.2021.19 PMC8959835

[31] Haslam A , Prasad V . Long COVID clinics and services offered by top US hospitals: an empirical analysis of clinical options as of may 2023. BMC Health Serv Res. 2024;24(1):684. doi:10.1186/s12913-024-11071-3 38816726 PMC11138016

[32] Ducharme J . Long COVID. Patients Wait Months for Treatment. Time. February 3, 2022. Accessed September 24, 2024. https://time.com/6144427/long-covid-treatments-health-care-wait/

[33] MacEwan SR , Rahurkar S , Tarver WL , et al. Patient experiences navigating care coordination for long COVID: a qualitative study. J Gen Intern Med. 2024;39:1294‐1300. doi:10.1007/s11606-024-08622-z 38308155 PMC11169119

[34] Graves J et al. From empathy to compassion fatigue: a narrative review of implications in healthcare. Empathy ‐ Advanced Research and Applications. IntechOpen; 2023.

[35] Amano A , Makowski MS , Trockel MT , et al. A qualitative study of strategies to improve occupational well‐being in physical medicine and rehabilitation physicians. Am J Phys Med Rehabil. 2024;103:674‐684. doi:10.1097/PHM.0000000000002555 38838100

[36] Rollins AL , Eliacin J , Russ‐Jara AL , et al. Organizational conditions that influence work engagement and burnout: a qualitative study of mental health workers. Psychiatr Rehabil J. 2021;44(3):229‐237. doi:10.1037/prj0000472 33793289 PMC8440452

[37] Solms L , van Vianen AEM , Koen J , et al. Physician exhaustion and work engagement during the COVID‐19 pandemic: a longitudinal survey into the role of resources and support interventions. PLoS One. 2023;18(2):e0277489. Published 2023 Feb 1. doi:10.1371/journal.pone.0277489 36724165 PMC9891506

